# Sensitivity of quantitative symmetry measurement algorithms for convergent beam electron diffraction technique

**DOI:** 10.1186/s42649-021-00060-z

**Published:** 2021-07-03

**Authors:** Hyeongsub So, Ro Woon Lee, Sung Taek Hong, Kyou-Hyun Kim

**Affiliations:** 1 Korea-Russia Innovation Center, Korea Institute of Industrial Technology, Incheon, 22004 Republic of Korea; 2 Department of Materials and Science Engineering, Korea University, Seoul, 02841 Republic of Korea

**Keywords:** Convergence beam electron diffraction, Symmetry, Transmission electron microscopy

## Abstract

We investigate the sensitivity of symmetry quantification algorithms based on the profile R-factor (*R*_*p*_) and the normalized cross-correlation (NCC) coefficient (*γ*). A DM (Digital Micrograph^©^) script embedded in the Gatan digital microscopy software is used to develop the symmetry quantification program. Using the Bloch method, a variety of CBED patterns are simulated and used to investigate the sensitivity of symmetry quantification algorithms. The quantification results show that two symmetry quantification coefficients are significantly sensitive to structural changes even for small strain values of < 1%.

## Introduction

Convergent beam electron diffraction (CBED) with transmission electron microscopy (TEM) is a powerful tool to extract submicron information (Zuo et al. 1999; Zuo et al. 1989; Zuo 1998). Especially, local symmetry determination with the CBED technique is of importance in terms of understanding fine structures as regions of interest scale down to submicron scale. The general procedure for symmetry determination with the CBED technique follows from investigation of the zero-order Laue zone (ZOLZ) details in the zone axis pattern (ZAP) (Buxton et al. 1976). Symmetry in the ZOLZ CBED pattern is classified into ten two-dimensional (2D) point groups (Buxton, et al. 1976; Loretto 1994). The symmetry elements of the rotational or mirror develop point groups by generating regularly repeated constituents from a reference motif. The generated motifs have a specific pattern, which is called rocking curve information (or intensity profile). The 2D point group determination in the ZOLZ CBED pattern is based on the regularly repeated constituents. The obtained 2D point group is then used to specify the projection diffraction groups and possible diffraction groups which, in turn, determine the point group (Buxton, et al. 1976; Loretto 1994).

The symmetry recorded in the CBED patterns is in general determined using direct visual inspection. In many practical applications, however, experimental CBED patterns often contain uncertainty of rocking curve information. The uncertainty may stem from either experimental error or the original structure. The deviation can be ignored or taken into account for symmetry determination. In consequence, the symmetry recorded in the CBED pattern can be interpreted in different ways.

Recently, Kim et al. proposed a symmetry quantification method for CBED patterns using the profile *R*-factor (*R*_*p*_) (Jansen et al. 1994; Toby 2006) and the normalized cross-correlation coefficient (*γ*) (Lewis 1995) to revel symmetry breaks and nanodomain structures in piezoelectric material (Jeon and Kim 2020). *R*_*p*_ and *γ* have been widely used to numerically calculate the degree-of-agreement between two objects. First, the profile *R*-factor (*R*_*p*_) has been used in Rietveld refinement to quantify the correlation between an experimental and computed intensity profile (Jansen, et al. 1994; Toby 2006). The normalized cross-correlation coefficient (*γ*) is another numerical measure to quantify the correlation between two image templates. The *γ* value is 1 (or 100%) when two image plates (symmetric CBED discs) are identical. In contrast, the *γ* value gets close to − 1 (or − 100%) as the amount of symmetry difference increases. The CBED disc consists of an intensity profile, so *R*_*p*_ can be used to compare the degree-of-agreement between two motifs. The whole disc image can also be considered as an image template. In this perspective, *γ* can be used to measure the amount of degree-of-agreement between the selected discs. The use of the symmetry quantification method, therefore, provides a more precise way to determine the symmetry in a CBED pattern. It was also proposed that the symmetry quantification method should be combined with a scanning electron diffraction technique for symmetry mapping (Tao et al. 2009; Zuo and Tao 2011).

In this study, we investigate the sensitivity of symmetry quantification methods for *R*_*p*_ and *γ* (NCC). Each algorithm is applied to several simulated structures such as strained Si and a perovskite structure to investigate the sensitivity of mathematical methods for symmetry quantification. This is because the experimental CBED patterns have unavoidable background noise generated by a CCD camera or inelastic scattering induced by something such as phonon vibration. For the simulation of CBED patterns, this study uses a Bloch wave method based on atomic scattering factors of Doyle and Turner (Doyle and Turner 1968) and the absorption parameters of Bird and King (Bird and King 1990).

## Methods of quantitative symmetry measurement

The symmetry quantification algorithms are embedded as DM (Digital Micrograph^©^) script. As proposed by Kim et al. (Kim and Zuo 2013), the developed algorithms consist of (1) disc selection, (2) alignment, (3) application of symmetry operation, and (4) symmetry quantification. Details on the imaging process for symmetry quantification have been reported elsewhere (Hu et al. 2000).

For the symmetry quantification of a CBED pattern, *R*_*p*_ is modified to quantify the similarity between two selected CBED discs, as in the following,

1 $$ {\mathrm{R}}_{\mathrm{p}}=\sqrt{\frac{\sum {\left\{{\mathrm{I}}_{\mathrm{B}}\left(\mathrm{x},\mathrm{y}\right)-{\mathrm{I}}_{\mathrm{A}}\left(\mathrm{x},\mathrm{y}\right)\right\}}^2}{\sum {\mathrm{I}}_{\mathrm{A}}{\left(\mathrm{x},\mathrm{y}\right)}^2}} $$

where I_A_(x, y) and I_B_(x, y) are the intensities of the selected CBED discs A and B at the address of (x, y) in pixels, respectively. Because the two selected templates are similar, the intensity difference sum between selected CBED discs A and B approaches zero, so that the smaller *R*_*p*_ value provides the better match. The two selected CBED discs will be referred to as image templates A and B. The DM script is then expressed as follows to calculate the *R*_*p*_ value.

Number RProfileFactor(Image ImgA, Image ImgB)

{

Number Numerator, Denominator

Numerator = sum((ImgB-ImgA)**2)

Denominator = sum(ImgA**2)

return sqrt(Numerator/Denominator)

}

The normalized cross-correlation (NCC) coefficient, *γ*, basically follows from the sum of the multiplication of differences between the image template and the mean of the image template for two templates. The cross-correlation coefficient is then normalized as follows,

2 $$ y=\frac{\sum_{\mathrm{x},\mathrm{y}}\left\{\left[{\mathrm{I}}_{\mathrm{A}}\left(\mathrm{x},\mathrm{y}\right)-{\mathrm{I}}_{\mathrm{A}}\right]\times \left[{\mathrm{I}}_{\mathrm{B}}\left(\mathrm{x},\mathrm{y}\right)-{\mathrm{I}}_{\mathrm{B}}\right]\right\}}{\sqrt{\left\{{\sum}_{\mathrm{x},\mathrm{y}}{\left[{\mathrm{I}}_{\mathrm{A}}\left(\mathrm{x},\mathrm{y}\right)-{\mathrm{I}}_{\mathrm{A}}\right]}^2\times {\sum}_{\mathrm{x},\mathrm{y}}{\left[{\mathrm{I}}_{\mathrm{B}}\left(\mathrm{x},\mathrm{y}\right)-{\mathrm{I}}_{\mathrm{B}}\right]}^2\right\}}} $$

where $\overline{\mathrm{I}}$_A_ and $\overline{\mathrm{I}}$_B_ are the mean values of two templates (Lewis 1995). In the Eq. (2), the numerator and denominator have exactly the same values if the two templates are absolutely identical. In contrast to *R*_*p*_, the cross-correlation coefficient is close to 1 when the two templates are identical. For a symmetry quantification algorithm based on *γ*, the DM script can be written as follows.

Number CrossCorrelation(Image ImgA, Image ImgB).

{

Number Numerator, Denominator.

Numerator = sum((ImgA-mean(ImgA))*(ImgB-mean(ImgB))).

Denominator = sqrt(sum((ImgA-Mean(ImgA))**2)*sum((ImgB-mean(ImgB))**2)).

return Numerator/Denominator.

}

## Applications and discussion

### Strained Si

Strained Si is attractive as a potential structure for advanced complementary metal-oxide-semiconductor (CMOS) technology or electro-optic devices (Erdtmann and Langdo 2006; Jacobsen et al. 2006). The amount of strain rate is very small, at a few percentage points, and is difficult to detect for local areas using X-ray diffraction technique due to the relatively large beam probe size of X-ray (Kim and Zuo 2014). An Si single crystal has a space group of $Fd\overline{3}m$ with lattice parameters of *a* = *b* = *c* = 5.4309 Å and with atomic coordinates of Si (0, 0, 0). The original structure of Si is then artificially strained along the [100]_C_, [010]_C_, and [001]_C_ directions by (0.5%, 0.5%, − 0.25%), (1%, 1%, − 0.5%), and (2%, 2%, − 1%). Hereinafter, the degree of applied strain will be referred to as *ε*_0_, *ε*_I_, *ε*_II_, and *ε*_III_ for (0%, 0%, 0%), (0.5%, 0.5%, − 0.25%), (1%, 1%, − 0.5%), and (2%, 2%, − 1%), respectively. The strain rates were determined based on the usual amount observed in the CMOS device. In the strained Si structure, a zone axis is properly selected to observe the effect of strain on the symmetry breaking in the recorded pattern symmetry. For example, the symmetry element along [100]_C_ is more sensitive to the applied strain than is that of [111]_C_. This is because the symmetry element is projected onto a two-dimensional CBED pattern along the observing direction. Thus, CBED patterns are simulated for zone axes of [100]_C_ and [111]_C_ to quantify the pattern symmetry based on the proposed symmetry quantification algorithms.

**Fig. 1:**
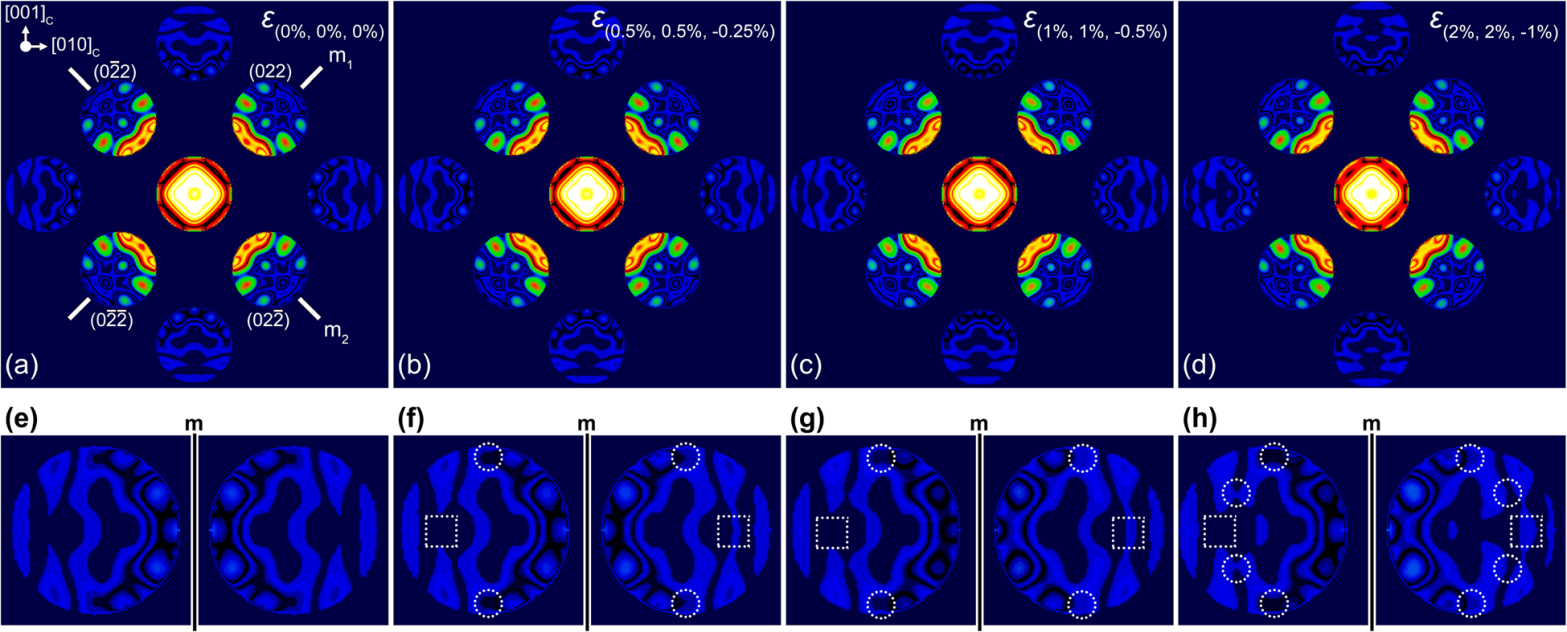
Calculated CBED patterns for the perfect Si struct. and (**b**-**d**) the strained Si structures at the zone axis of [100]_C_. The amount of strain is denoted in each simulated CBED patterns

Figure 1a shows the simulated CBED patterns of Si for the zone axis of [100]_C_ at the thickness of 60 nm. The pattern symmetry of Si has *4 mm* at the zone axis of [100]_C,_ as shown in Fig. 1a. Figures 1a-d show the simulated CBED patterns for the strains of *ε*_I_, *ε*_II_, and *ε*_III_. Overall features of simulated CBED patterns are very similar for different amounts of strain while the amount of strain increases from *ε*_I_ to *ε*_III_. Differences in the rocking curve information are only observable in the magnified discs images. Figure 1f, for example, shows magnified CBED discs of (004) and (040)_m_, where ‘m’ indicates that the mirror symmetry is applied to the (040) disc. In the magnified image with *ε*_I_, the two CBED discs have considerably small differences in the rocking curve information, as indicated by the dotted circle and the rectangle. Similarly, other simulated CBED patterns (Figs. 1g and h) for the larger strain values also induce very small changes in the rocking curve information; these changes are very hard to determine only by visual inspection.

**Fig. 2:**
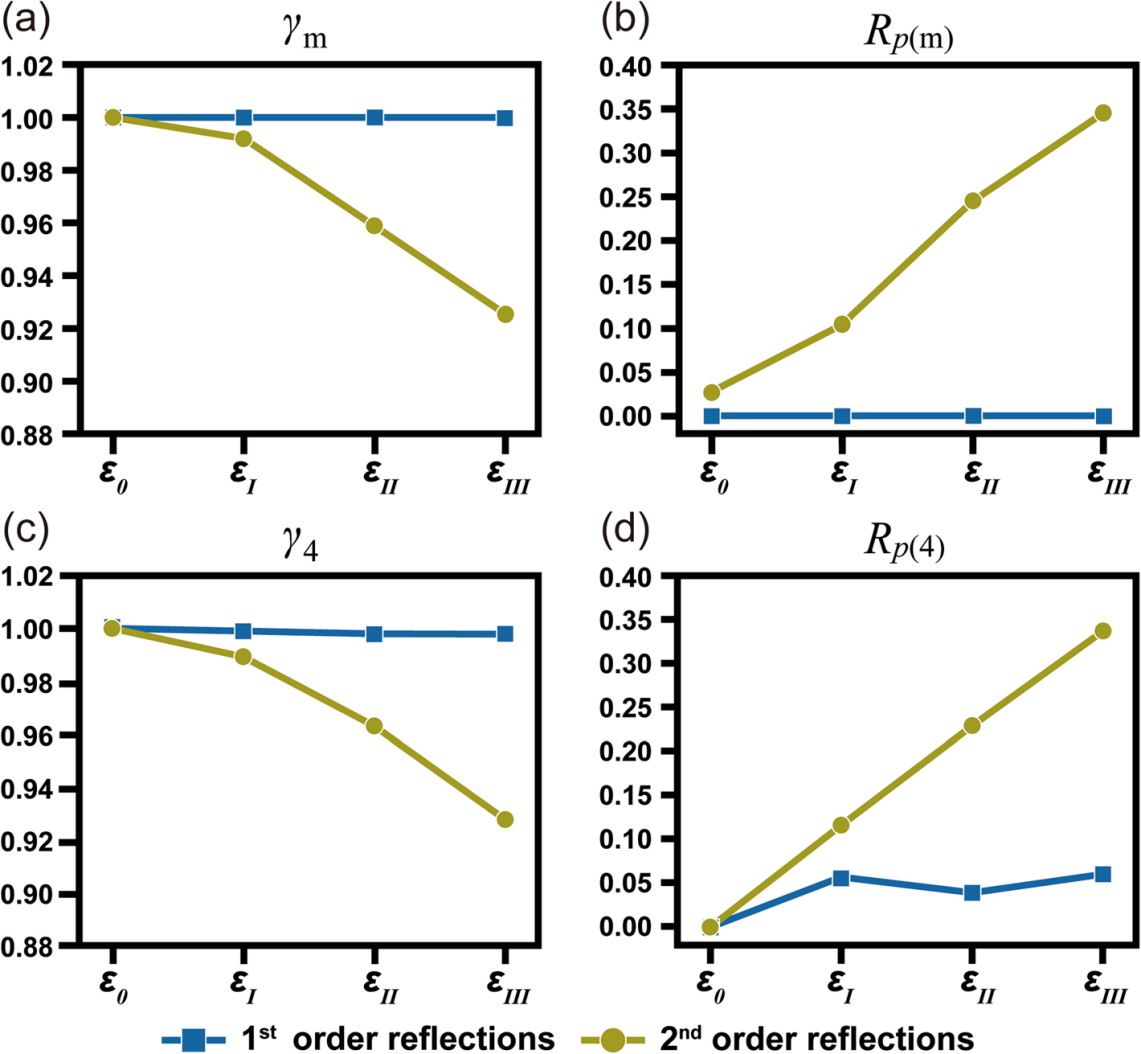
Symmetry quantification for mirror and 4-fold rotational symmetry recorded in the simulated CBED patterns for *ε*_0(0%, 0%, 0%)_, *ε*_I(0.5%, 0.5%, − 0.25%)_, *ε*_II(1%, 1%, − 0.5%)_, *ε*_III(2%, 2%, − 1%)_. The symmetry elements are respectively quantified based on *γ* and *R*_p_

Symmetry breaking induced by strain is then measured by quantitative methods based on the R-factor and the γ coefficient, as shown in Fig. 2. Symmetry measurements were performed for the 1st order reflections and for the 2nd order reflections. For mirror symmetry, the (022)/(0 $\overline{2}$ 2) and (0 $\overline{2}\overline{2}$)/(02 $\overline{2}$) discs along m_1_ are selected for 1st order reflections and the (004)/(040) and (00 $\overline{4}$)/(0 $\overline{4}$ 0) discs are selected for 2nd order reflections. The same discs sets are selected to calculate the 4-fold rotational symmetry. Figures 2a and b show the variations of mirror symmetry calculated by *γ*_m_ and *R*_*p*(m)_ for the 1st and the 2nd order CBED discs. For the 1st order CBED discs, the quantification values indicate that mirror symmetry is almost maintained while the amount of strain increases from *ε*_0_ to *ε*_III_. In contrast, it can be observed that mirror symmetry is obviously broken in the 2nd order reflections. The *γ*_m_ value decreases from 100% to 92.5% as the amount of strain increases. Also, the *R*_*p*(m)_ value increases from 0.028 to 0.346 as the amount of strain increases. The sensitivities to the structural change, however, are different between the applied algorithms. From the symmetry quantification results, the perfect mirror symmetry of *γ*_m_ (=100%) slightly decreases to 99.2%, 95.9%, and 92.5% for *ε*_I_, *ε*_II_, and *ε*_III_, respectively. In contrast, the *R*_*p*(m)_ value has 0.028 for the perfect Si structure and dramatically increases to 0.104, 1.245, and 0.346. By comparting the two algorithms, the *R*_p_ value shows an almost 15 times difference in the calculation results between *ε*_I_ and *ε*_II,_ while changes of only a few percentage points are only observed for the *γ* value. The *γ* value shows very similar results for the 4-fold rotational symmetry, as shown in Fig. 2c. The 4-fold rotational symmetry is almost maintained for the 1st order, while the quantification results for the 2nd order are affected by the strain. Unlike the results of the *γ* values, however, the *R*_*p*_ values vary from 0 ~ 0.061 for the 1st order. Nevertheless, *R*_*p*_ of *ε*_II_ has a smaller value than *ε*_I_ and *ε*_III,_ even though *R*_*p*(4R)_ values are expected to increase gradually as the amount of strain increases. This suggests that the *γ* coefficient is more correlated with the symmetry change than is *R*_*p*_, while the *R*_*p*_ is more sensitive to the symmetry change. In addition, the above results indicate that symmetry broken by structural distortion has a greater effect on symmetrical relation in the 2nd or higher order reflections than in the 1st order reflections.

**Fig. 3:**
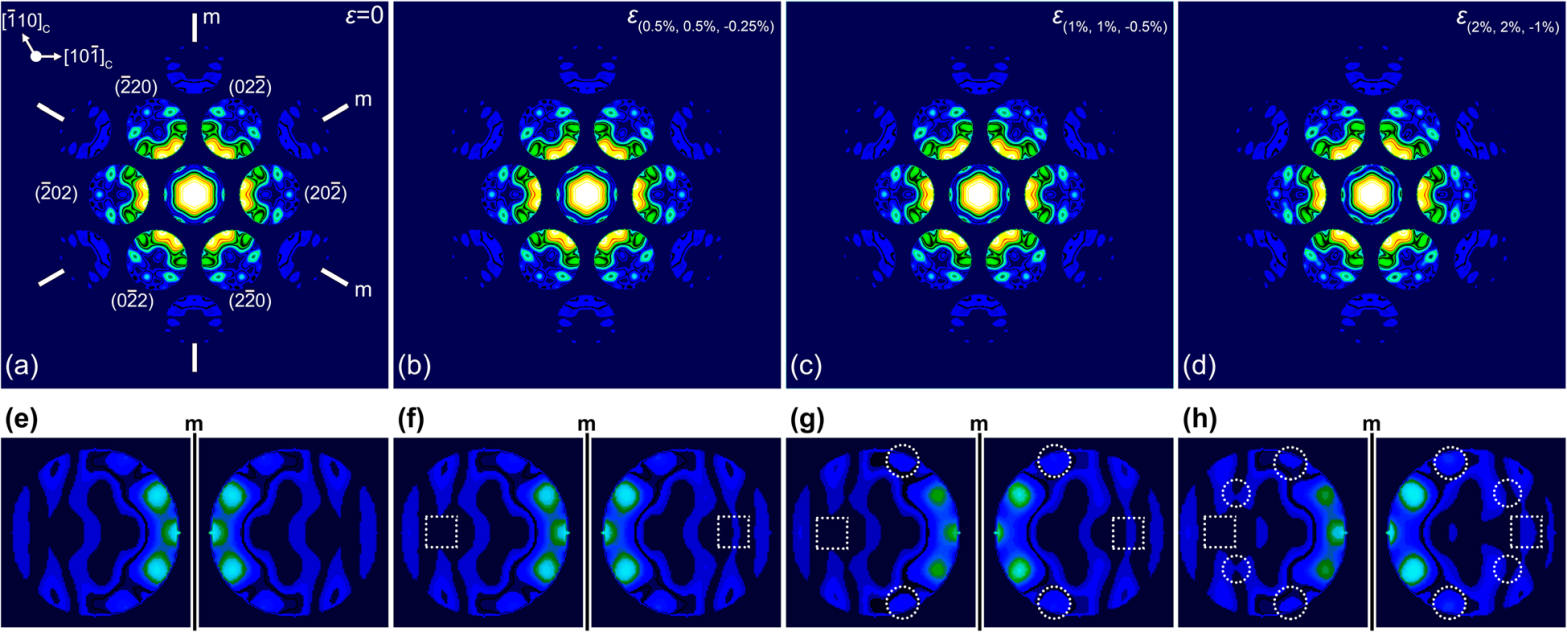
Calculated CBED patterns for the perfect Si structure and (**b**-**d**) the strained Si structures at the zone axis of [111]_C_. The amount of strain is denoted in each simulated CBED patterns

Figure 3 shows simulated CBED patterns for Si with different strain values. The CBED patterns were calculated for the sample thickness of 60 nm and for the strain values used in Figs. 2 and 3. Like the simulated [100]_C_ CBED patterns, the broken symmetry is hard to observe in the simulated CBED patterns by visual inspection alone, as shown in Figs. 3a-d. Differences in the rocking curve information are compared for the mirror symmetry using the magnified second order reflections of ($\overline{2}$ 4 $\overline{2}$)/(22 $\overline{4}$)_m_, as shown in Figs. 3f-h. Only small differences are observed even in the magnified CBED discs, as well as in the results from the zone axis of [100]_C_. The quantitative symmetry measurements then follow using *R*_*p*_ and the *γ* coefficient.

**Fig. 4:**
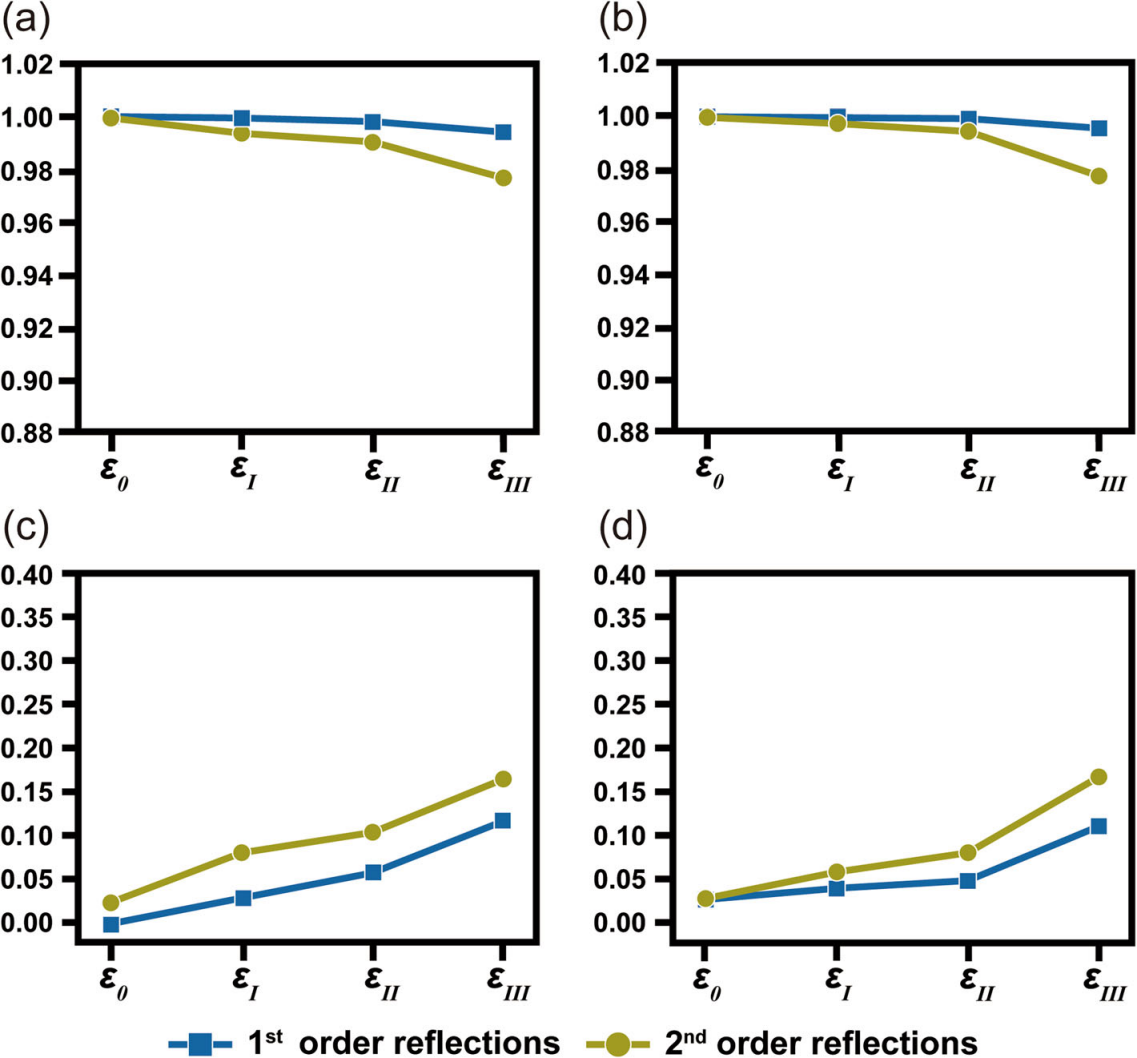
Symmetry quantification for mirror and 6-fold rotational symmetry recorded in the simulated CBED patterns for *ε*_0(0%, 0%, 0%)_, *ε*_I(0.5%, 0.5%, − 0.25%)_, *ε*_II(1%, 1%, − 0.5%)_, *ε*_III(2%, 2%, − 1%)_. The symmetry elements are respectively quantified based on *γ* and *R*_p_

Figure 4 shows the effect of strain on the deviation of the symmetry element along the zone axis of [111]_C_. The recorded symmetry is quantified for the mirror and for the 6-fold rotational symmetry based on the 1st order and 2nd order reflections, respectively. Similar to the results of [100]_C_, the quantification results indicate that the degree of broken symmetry gradually increases as the amount of applied strain increases. Nevertheless, the symmetry quantification values are not strictly related to the amount of applied strain. For example, the γ_m_ values for the second order reflections decrease from ~ 99.9% (*ε*_0_) to 99.3% (*ε*_I_) → 99.04 (*ε*_II_) → 97.7 (*ε*_III_). The calculated γ_m_ value abruptly decreases at *ε*_III_, while the applied strain values uniformly increase by two times for *ε*_I_ → *ε*_II_ → *ε*_III_. The *R*_*P*(m)_ values also dramatically increase at *ε*_III_ as well.

By considering the results from the zone axis of [100]_C_, quantitative symmetry measurement along the zone axis of [111]_C_ shows that the amount of broken symmetry is smaller than for the results along the zone axis of [100]_C_. The degree of symmetry broken by applied strain becomes small along the higher index of the zone axis because the pattern symmetry of CBED results from the projected atomic coordinates. Considering this, strain induced broken symmetry is more obviously observable along the zone axis of [100]_C_ than the zone axis of [111]_C_.

### Symmetry change in relaxor-based ferroelectric materials

Relaxor-based ferroelectric ceramics such as (1-x)Pb(Mg_1/3_Nb_2/3_)O_3_-xPbTiO_3_ (PMN-xPT) have attracted much research interest due to the strong dependence of their polarization on the applied electric field. A single crystal of PMN-xPT with low contents of PT (x < ~30%) has rhombohedral symmetry at room temperature, in which **P**_**S**_, a spontaneous polarization**,** is constrained to the [111] direction. In a rhombohedral composition near the morphotropic phase boundary (MPB) (Choi et al. 1989; Kim et al. 2012), PMN-PT achieves ultrahigh piezoelectric responses along the non-polar direction of [001], which leads to electric field-induced phase transition from R (rhombohedral, *R3m*) to T (tetragonal, *P4mm*) symmetry (Noheda 2002). Indeed, R and T symmetry are not allowed for direct phase transition. The polarization vectors then rotate within the mirror plane (Fu and Cohen 2000) common to the R and T symmetry. The R symmetry undergoes a structural change into T symmetry via the paths of ‘R → M (monoclinic, *Pm* or *Cm*) → T (Noheda 2002). Nevertheless, it is still unclear whether the giant piezoelectric properties stem from the observed monoclinic phases (Viehland 2000; Kisi et al. 2003). On the other hand, structural similarities among the known R, M, and T have been a key issue in determining the exact crystallographic information in PMN-xPT single crystals. The high temperature phase of cubic in PMN-xPT is transformed into R, M, or T with very small structural distortions, as listed in Table 1. In consequence, the conventional electron diffraction and X-ray diffraction techniques are hard to apply to identify the phase of PMN-xPT. The CBED patterns of PMN-PT with x = 31% are simulated for the reported crystallographic information and the pattern symmetry is quantified using *γ* and *R*_*p*_.

**Table 1:** Crystallographic information of PMN-31PT for cubic, monoclinic, rhombohedral, and tetragonal

Crystal system	Cubic	Monoclinic		Rhombohedral	Tetragonal
Space group	*Pm3m*	*Cm*	*Pm*	*R3m*	*P4mm*
Lattice parameters	*a* = *b* = *c* = 4.0191 *α* = *β* = *γ* = 90^o^	*a* = 5.6951 *b* = 5.6813 *c* = 4.0138 *α* = *γ* = 90^o^ *β* = 90.136^o^	*a* = 4.0183 *b* = 4.0046 *c* = 4.0276 *α* = *γ* = 90^o^ *β* = 90.146^o^	*a* = *b* = *c* = 4.0364 *α* = *β* = *γ* = 89.8826^o^	*a* = *b* = 3.9920 *c* = 4.0516 *α* = *β* = *γ* = 90^o^
Atomic coordinates	*Pb(0, 0, 0)* *TiNb/Mg(0.5, 0.5, 0.5)* *O(0.5, 0.5, 0)*	*Pb(0, 0, 0)* *TiNb/Mg(0.5250, 0, 0.498)* *O*_*1*_*(0.54, 0, −0.01)* *O*_*2*_*(0.317, 0.267, 0.48)*	*Pb(0, 0, 0)* *TiNb/Mg(0.509, 0.50, 0.5479)* *O*_*1*_*(0.47, 0, 0.57)* *O*_*2*_*(0.417, 0.5, 0.509)* *O*_*3*_*(−0.02, 0.5, 0.57)*	*Pb(0, 0, 0)* *TiNb/Mg(0.534, 0.534, 0.534)* *O(0.541, 0.541, 0.03)*	*Pb(0, 0, 0)* *TiNb/Mg(0.5, 0.5, 0.532)* *O*_*1*_*(0.5, 0.5, 0.054)* *O*_*2*_*(0.5, 0, 0.601)*

**Fig. 5:**
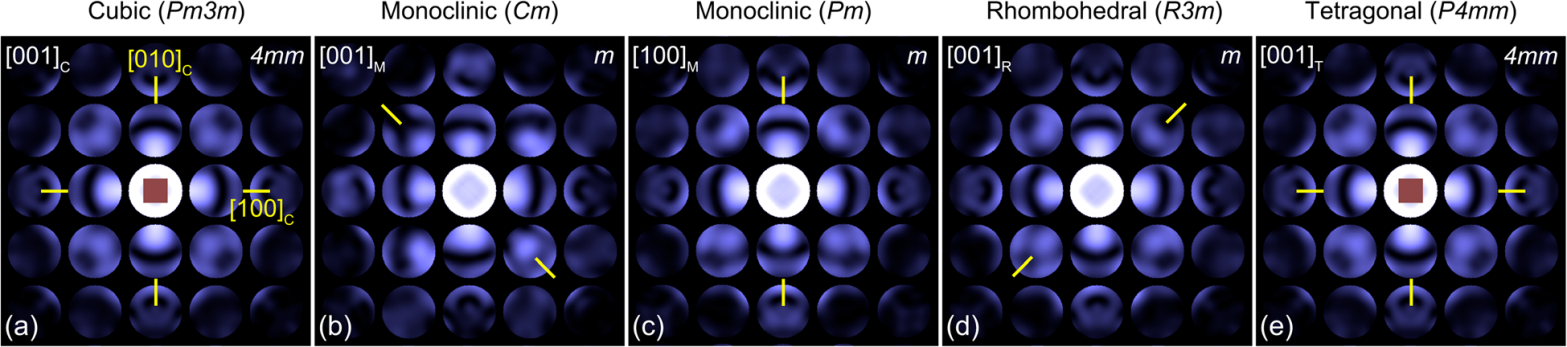
Simulated CBED patterns for (**a**) the zone axis of [001]_C_ (*Pm3 m*), (**b**) the zone axis of [001]_M_ (*Cm*), (**c**) the zone axis of [100]_M_ (*Pm*), (**d**) the zone axis of [001]_R_ (*R3m*), and (**e**) the zone axis of [001]_T_ (*P4mm*)

Figure 5 shows the calculated CBED patterns of the C, M (*Cm*, *Pm*), R, and T phases at the zone axis of [001]_C_ with respect to the pseudo-cubic axes at the sample thickness of 80 nm. For the pseudo-cubic axis of [001]_C_, the corresponding zone axes of M (*Cm*, *Pm*), R, and T phases are respectively [001]_M(*Cm*)_, [100]_M(*Pm*)_, [001]_R_ and [001]_T_. As shown in the simulated patterns, the pattern symmetries can be only ambiguously distinguished by visual inspection, while the CBED patterns are simulated with different crystal structures. For the zone axis of [001]_C_, the C, M (*Pm*, *Cm*), R, and T phases have pattern symmetries of *4mm*, *m*, *m*, *m*, and *4mm*, respectively, as indicated in Fig. 5. The mirror symmetry element in the CBED patterns is then selected for the symmetry quantification because the polarization vector, **P**_**S**_, in PMN-PT lies on the mirror plane of each phase. To see the amount of mirror elements, mirror symmetry is quantified along the directions of [100]_C_, [010]_C_, and [110]_C_ for each CBED pattern in order to see the variations of mirror symmetry elements subjected to structural distortions, i.e., phase transformation.

**Fig. 6:**
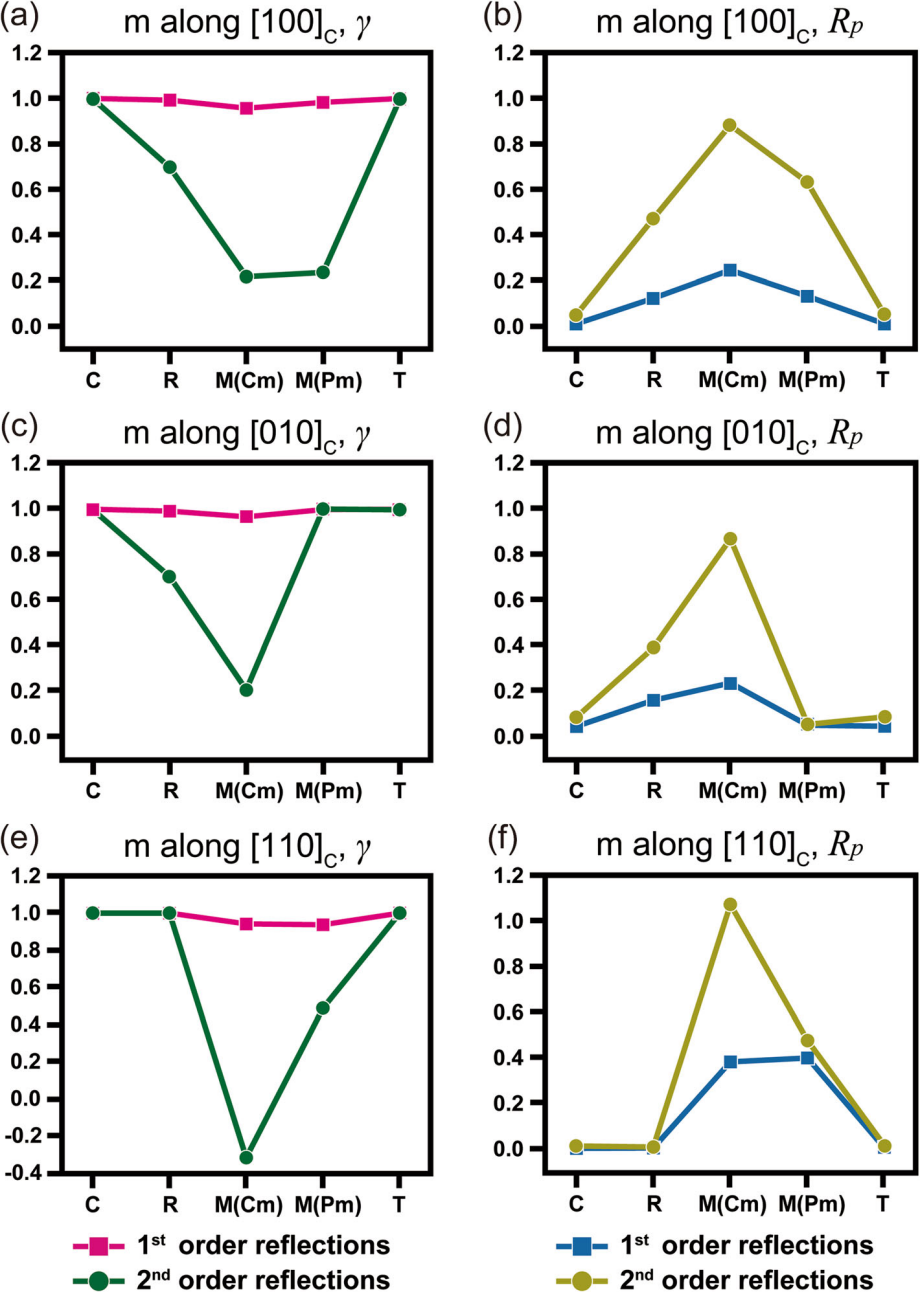
Quantified symmetry element for simulated CBED patterns of PMN-31%PT

Figure 6 shows profiles of mirror symmetry quantification results for the C, R, M (*Cm*), M (*Pm*), and T phases along the different directions of [100]_C_, [010]_C_, and [110]_C_. As shown in the quantification results, the *γ*_m_ values of the 1st order reflections are almost maintained for all directions. This indicates that, among the reported crystallographic information, the structural distortions induced by phase transformation are very small. Similarly, by considering the result of strained Si, the *R*_*p*_ values of the 1st order reflections are found to vary by small amounts. In contrast, broken symmetry is obviously observed in the 2nd order reflections. From the quantification results, the amount of mirror symmetry has the lowest value for the M phase of *Cm*. Then, the amount of mirror symmetry gradually increases. This is mainly due to polarization rotation. In a previous study, it was found that the polarization vector rotates from R ([111]_C_) to T ([001]_C_) via the monoclinic phases of *Cm* and *Pm*. On the pseudo-cubic axes, the polarization vector rotates along (100)_C_ of M (*Cm*) → (010)_C_ of M (*Pm*). It is well known that phase transformation occurs via C → R → M (*Cm*) → M (*Pm*) → T (*P4mm*). Based on that previous report, the phase transformation from R to M (C*m*) is subjected to the first order transition, while other transformation steps follow by second order transition. From a structural aspect, the rhombohedral crystal structure requires severe distortion to transform into monoclinic axes, as listed in Table 1. In consequence, the drastic γ changes between R and M (C*m*) are considered as the degree of amount of structural change for the phase transition between R → M.

## Conclusion

We investigated the sensitivity of two symmetry quantification coefficients of the profile *R*-factor (*R*_*p*_) and the normalized cross-correlation coefficient (*γ*). The quantification results show that the two different coefficients are significantly sensitive to deviation of the symmetry element. Only the few strain values of < 1% are obviously detected by *R*_p_ and the *γ* coefficient. In addition, small structural distortions can also be evidently differentiated using the two coefficients. Nevertheless, the two coefficients *R*_p_ and *γ* show differences in application to symmetry quantification. Compared to *γ*, *R*_p_ shows huge differences for small structural changes, so it is more applicable to visualizing small differences in a crystal structure. Because it is not normalized, however, *R*_*p*_ cannot be used to compare CBED patterns recorded from different samples. Moreover, in some cases, *R*_*p*_ only uncertainly shows the amount of symmetrical change, as demonstrated in Fig. 2d. In comparison, because it is normalized, the *γ* value can be directly applied to quantify the symmetry elements recorded in different CBED patterns. Due to its normalization, the *γ* value is not affected by different operation conditions. Also, the *γ* value exactly agrees with the structural change in all cases.

## Data Availability

Not applicable.
